# Relationship between the cost of illness and quality of life among adolescents with type 1 diabetes—a mixed method study

**DOI:** 10.1038/s41598-024-63536-4

**Published:** 2024-06-11

**Authors:** Sulochanadevi B. Chakrashali, B. Madhu, M. Mounika Sree, M. Chaithra, K. S. Sahana, K. Nagendra

**Affiliations:** 1 School of Public Health, JSS Medical College, JSS Academy of Higher Education & Research, Mysuru, Karnataka India; 2 Department of Community Medicine, JSS Medical College, JSS Academy of Higher Education & Research, Bannimantap, Mysuru, Karnataka 570015 India; 3 Department of Community Medicine, JSS Medical College, JSS Academy of Higher Education & Research, Mysuru, Karnataka India; 4 Department of Paediatrics, Mysore Medical College & Research Institute, Mysuru, Karnataka India

**Keywords:** Type 1 diabetes mellitus, Cost of illness, Diabetes-specific quality of life, Adolescents, Type 1 diabetes, Diseases, Health care

## Abstract

Type 1 diabetes mellitus (T1DM) is a major problem worldwide that affects the quality of life, well-being of patients and their families. This study aimed to determine the relationship between the cost of illness and quality of life among patients with T1DM. A concurrent, parallel, mixed-method study of 113 adolescents with T1DM registered in public and private hospitals in the Mysore district was conducted by obtaining data related to the cost of illness and quality of life using a validated Diabetes-Specific Quality of Life (DSQoL) questionnaire. Thematic analysis was used to identify the themes. There was a significant association amonghealth insurance status, treatment facility type, catastrophic health expenditure (CHE), and cost of illness. The CHE proportion was32.7%. Financial sources for treatment were met primarily by borrowing money with interest (58 patients, 51.3%), followed by individualincome (40 patients, 35.3%), contributions from friends and relatives (10 patients, 8.8%), and selling of assets (5 patients, 4.4%). The monthly health expenditures of approximately 22 (19.46%) households were greater than their monthly incomes. There was a positive correlation (rvalue of 0.979) between the cost of treatment and the DSQoL score, and this correlation was statistically significant, with a *p* value < 0.001. The higher theDSQoL score was, the worse the quality of life and the worse the well-being of T1DM patients. Three themes were identified: the impact of financial cost on family coping, the impact of financial cost on seeking care and the emotional burden of financial cost. There was a statistically significant positive correlation between the cost of treatment and the DSQoLscore. Adolescents with T1DM who had greatertreatment costs had worseDSQoL, and significantly lower health expenses were observed among adolescentswho had health insurance. Cost of illness acts as a barrier to treatment and placesa burden on patients and their families.

## Introduction

Type 1 diabetes mellitus (T1DM is a major problem worldwide that affects the psychosocial, economic, and emotional well-being of patients and their families^1^. According to the International Diabetes Federation's recent publication (2011), 366 million adults are living with diabetes,accounting for 8.3% of the adult population worldwide. It is estimated that 552 million or 9.9% of adults will have diabetes by 2030^2,3^.

T1DM can lead to heart disease, blindness, hypoglycemia, hyperglycemia, kidney failure, diabetic ketoacidosis, psychiatric illness, and hearing loss. T1DM increases the risk of early death and decreases quality of life^2,4^. It is estimated that the median direct cost of diabetes placesa very high economic burden on individuals with T1DM and households^5^. Patients spend more than 16% of their income on outpatient care, 23% of their income on treatmentwhen hospitalized,leading to catastrophic health expenditures^6^. The lifetime economic burden of T1DM patients in the United States is $813 billion (95th percentile range $682 to $1037 billion), which represents a high economic burden on T1DM patients^7^.

Glycemic control and the cost of treatment were found to be negatively correlated. Furthermore, a lower cost of care was associated with a high burden of psychosocial illness and poor quality of life^8^. It was found that taking medicine is not a problem for patients, but arranging for the money to pay for treatment is a major problem. T1DM takes away all the things that make life enjoyable for patients and has become a burden forindividuals and households;adolescents with T1DM need five times more care and support when compared to healthy adolescents^9^. Endocrinologists and experts opine that treatment adherence can help a T1DM patientbe healthy, but there is no cure for T1DM;therefore, these patientshave to take medicine for the rest of their lives.^10^**.**

Thus, improving the quality of care and reducing the financial burden can help T1DM patients achieve a better quality of life. There is limitedevidence onlocal cost and QOL; thus, this study aimed to determine the economic burden and the relationship between the cost of illness and diabetes-specific quality of life among adolescents with T1DM in Mysore and to assess whether cost acts as a barrier to treatment.

This study addresses the pressing issue of T1DM), which significantly impacts global health and imposes a substantial economic burden. Despite thesevere health implications of T1DM, including various complications and reduced quality of life, there is limited local evidence on the economic burden and its relationship with quality of life among adolescents with T1DM in Mysore. Understanding this relationship is crucial for improving care quality, alleviating financial strain, and enhancing the well-being of affected individuals and their families.

### Objectives

1. To determine the associations between the economic burdens of direct out-of-pocket health expendituresand sociodemographic and economic variablesin households with adolescents with type 1 diabetes.
2. To estimate the proportion of households experiencing catastrophic health careexpenditure.
3. To explore whether financesources and costsarebarriersto treatment among adolescents with type one diabetes.
4. To determinethe relationship between the cost of illness and diabetes-specific quality of life.

## Methodology

### Study design

A concurrent, parallel, mixed-method study design was used to investigate the factors influencing quality of life among adolescents with type 1 diabetes mellitus (T1DM).

### Study area

The study was conducted in Mysuru, Karnataka, India. The data collection took place in one public hospital, one private tertiary care hospital and three endocrinology clinics in the city where T1DM patients were available.

Sampling Technique: Convenience sampling was used at health facilities, with participants universally selected from registers within the 10–19 years age group. We obtained details on the adolescents’health facility visits, obtained assent and consent from guardians for their participation.

### Study population

The study population consisted of adolescents with T1DMwho were registered in the selected health care facilities of Mysurubetween January 2021 and January 31, 2022.

### Sample size

The sample size was calculated based on the prevalence data provided by Das A K, ^11^ which indicated a prevalence of 17.93 T1DM cases per lakh children in Karnataka. Assuming a prevalence of 17.93%among known T1DM patients, with an absolute allowable error of 7% and a confidence level of 95%, the required sample size for the study was calculated to be 111 participants(formula: Zαpq/d^2^).

### Inclusion criteria

The inclusion criteria were adolescents between the ages of 10 and 19 years who had been diagnosed with T1DM for at least one year and volunteered to participate in the study with their parent's or guardian's consent.

### Exclusion criteria

The exclusion criteria were adolescents diagnosed with type 2 diabetes, those with mental retardation or severe psychological illness, and those who were unable to complete the interviews or unwilling to provide assent/consent.

### Data collection

Data collection was performed using a combination of in-person interviews (72%) and phone interviews (28%). In-person quantitative interviews were conducted during the adolescents' follow-up visits to the hospital. For adolescents who wereunable to attend the follow-up visits, interviews were conducted over the phone using Google forms. Among 72% (80 participants) in person interview, only 15 (patients or caregivers) dedicated extra time to participate in in-depth interviews, providing rich qualitative data.

The data collection period spanned from January 2021 to January 31, 2022.

### Study variable definitions

**I) Socioeconomic status** was determinedaccording to the Kuppuswamy Socioeconomic Scale-2019.^12^**.**

**ii) Diabetes status** was recorded according to the WHO criteria:^13^**.**

- Controlled—Patients with an RBS level > 200 mg/dl,an FBS level > 126 mg/dl or HbA1clevel > 6.5%;
- Not controlled—Patients with an RBS level < 200 mg/dl,an FBS level < 126 mg/dl or an HbA1clevel < 6.5%^3^
- Unknown—Patients for whom the present diabetes status was not known.

iii) Education level:

Primary school—5th to 7thstandard

High school—8th to 10th standard

Preuniversity college—11th and 12th standard

## iv) Health Facilities

- **Private Tertiary Care Hospital (JSS Hospital)**: A privatelyowned advanced medical facility, free medications were given for T1DM.
- **Public Tertiary Care Hospital (Government K R Hospital)**: A government-funded medical facility providing specialized care to the public**.**
  o **Private Clinics**: Outpatient facilities where patients receive consultations and treatments by appointment.
  o **Private Hospitals**: Privatelyowned advanced medical institutions where services typically incur costs without free provisions.

**v) Outpatient Care cost:** All the costs were calculated based on the statements of the adolescents’ parents or guardians.

**Direct medical costs** reported for the previous month included the fees paid for physicians consultations, fees paid for laboratory tests, and medicine costs.

**Direct nonmedical costs**werecalculated for travel expenses and food expenses for both the patient and the person who accompanied them for T1DMtreatment.

## vi) Inpatient care (Hospitalization):

T1DM patients who were admitted to the hospital solely for diabetes-related concernsand stayed overnight for treatment in the last 12 months were included, and reported yearly expenditures were changed to monthly expenditures before the total monthly expenditure was computed.

vii) To determine whether **cost acted as a barrierto treatment,** we asked whether there was any timein the previous 12 months when the patients were not able to take amedicationrecommended by aphysician or had a problem paying physician or staff nurse fees, laboratory fees, or any hospital fees.

viii) If the total health expenditure of a family exceeded10% of their annual income, then thefamily wasdeemed to have had**catastrophic health expenditures**.^14^.

ix) If the total health expenditure of a family pushedthe family below the official poverty line, then thefamily wasdeemed to have been **impoverished** because of medical reasons.^14^**.**

### Ethical considerations

The study obtained ethical clearance and was approved by the Institutional Ethical Committee (JSSMC/IEC/05,012,022/37NCT/22,021-22 DATE 14-1-2022) of JSS Medical College. All methods were performed in accordance with the relevant guidelines and regulations. Written informed assent/consent was obtained from all participating adolescents and their parents or guardians before data collection.

### Study tools

- **Sociodemographic Questionnaire:** This questionnaire collected sociodemographic information from the study participants, including age, sex, educational level, family income, and other relevant demographic variables.
- **Diabetes-Specific Quality of Life (DSQoL) Questionnaire:** The DSQoL questionnaire is a validated instrument designed to assess quality of life specifically related to T1DM. The 64-item DSQoLwas designed specifically for people with T1DM . It includes 44 burden items measuring the impact of diabetes on “social relations,” “leisure time flexibility,” “diet restrictions,” “physical complaints,” “daily hassles,” and “worries about the future.” Respondents are asked to rate the extent to which each of the statements meets their “point of view” on a 6-point Likert scale from 5 = “perfectly” to 0 = “not at all.” An additional 10 items measure treatment satisfaction (scored on a 6-point scale from 0 = “very satisfied” to 5 = “very dissatisfied”), and 10 other itemsassess the personal importance of treatment goals (scored on a 6-point Likert scale from 5 = “very important” to 0 = “totally unimportant”).^15^The formula “total score/320*100”is used toobtain the QOL score (%);a higher percentageindicates worsediabetes-relatedquality of life and worse well-being.

### Data analysis

The collected data were coded and entered into a Microsoft Excel 2010 spreadsheet. Statistical analysis was performed using SPSS version 24 software(licensed to JSS AHER). Descriptive statistical measures, such as the frequency and percentage the median and interquartile range,were calculated. As the data were not normally distributed, nonparametric tests, such as the Mann‒Whitney test, Kruskal–Wallis test, and Spearman correlation test, were employed to explore the relationships between variables (Tables 2, 3, 4). Linear regression was performed to determine the relationship between the cost of illness and quality of life. The results are presented in tables and graphs as appropriate. A p value less than 0.005 indicated statistical significance. The qualitative data were analyzed manually by thematic analysis categorizing quotes under specific themes.

## Results

**Figure 1:**
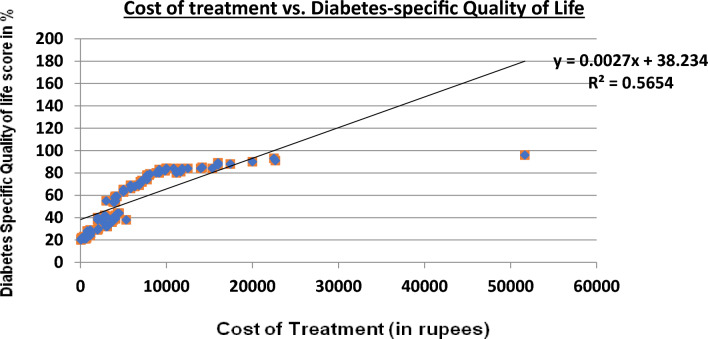
Correlation between the cost of illness and Diabetes specific Quality of life.

A total of 113 participants (N = 113) with T1DM were enrolled, and their socioeconomic and demographic characteristics are shown in Table 1. The associations between socioeconomic, demographic variables and outpatient and inpatient treatment costs are shown in Tables 2 and 3, respectively. Table 4 showsthe financial sources met, and the relationship between cost and quality of life is shownin Fig. 1.

**Table 1:** Socioeconomic and demographic characteristics of the study participants.

Characteristics	Categories	Number of participants (%)	
Adolescent Age Group (in years)	Early	10–13	52 (46%)
	Middle	14–17	34 (30%)
	Late	18–19	27 (24%)
Sex- Male	Early	10–13 Years	23 (20.3%)
	Middle	14–17 Years	23 (20.3%)
	Late	18–19 Years	13 (11.5%)
Female	Early	10–13 Years	29 (25.6%)
	Middle	14–17 Years	11 (9.7%)
	Late	18–19 Years	14 (12.3%)
Education Level of Head of the Family	Professional		3 (2.6%)
	Graduate		22 (19%)
	Intermediate or diploma		11 (9%)
	High School		27 (24%)
	Middle School		24 (21%)
	Primary school		8 (7%)
	Illiterate		18 (16%)
Socioeconomic Class^12^	Upper		1 (0.88%)
	Upper middle		11 (9.73%)
	Lower middle		36 (31.85%)
	Upper lower		59 (52.21%)
	Lower		6 (5.3%)
Health Insurance status	Insured		13 (11.5%)
	Uninsured		100 (88.4%)
No of years since initial diagnosis	> 1–4 years		89 (79%)
	5–8 years		18 (16%)
	9–12 years		6 (5%)
Level of glycemic control	Controlled		98 (86%)
	Not controlled		12 (11%)
	Don’t know		3 (3%)
Education level of the patient	Primary school		54 (48%)
	High school		36 (32%)
	Pre-university College		23 (20%)

**Table 2:** Association between socioeconomic and demographic characteristics and the cost of outpatient careamong participants.

Variable	Categories	Frequency (%)	OPD cost (Median-IQR)/month	OPD cost in USD $	*p* value
Area	Rural	54 (47.3)	3000(400–6250)	36.4 (4.8–75.9)	0.914
	Urban	59 (52.2)	3000(2000–3500)	36.4 (24.3–42.5)	
Type of Treatment Facility	Private Tertiary Care Hospital	58 (51.3)	2000(300–3000)	24.3 (3.6–36.4)	**0.004**
	Government-funded Tertiary Care Hospital	47 (41.5)	3000(2000–5000)	36.4 (24.3–60.7)	
	Private clinics/Hospital	8 (7.07)	5000(4000–10,000)	60.7 (48.6–121.5)	
Experiencing Catastrophic Health Expenditure	Yes	37 (32.7)	3000 (2000–5750)	36.4 (24.3–69.8)	0.735
	No	76 (67.2)	2500 (1500–3000)	30.3 (18.2–36.4)	
Socioeconomic Class (SEC)^11^	Upper	1 (0.88)	2000	24.3	0.496
	Upper middle	11 (9.73)	3000(2000–10,000)	36.4 (24.3–121.5)	
	Lower middle	36 (31.8)	3000(2000–3000)	36.4 (24.3–36.4)	
	Upper lower	59 (52.2)	3000(2000–5000)	36.4 (24.3–60.7)	
	Lower	6 (5.3)	4000(1500–9088)	48.6 (18.2–110.4)	
Health Insurance status	Insured	13 (11.5)	2500 (450–5000)	30.39 (5.47–60.7)	**0.008**
	Uninsured	100 (88.4)	3000 (2000–5000)	36.4 (24.3–60.7)	

* July 5 2023 exchange rate—82.3 INR₹/1 USD$

Significant values are in [bold].

**Table 3:** Association between socioeconomic and demographic characteristics and the cost of inpatient careamong participants.

Variable	Categories	Frequency (%)	IPD cost (Median-IQR)/year	IPD cost in USD	*p* value
Area	Rural	39 (46.4)	25,000 (100,000–50,000)	303.5 (1214.2–607.1)	0.173
	Urban	45 (53.6)	40,000 (20,000–50,000)	485.69 (242.8–607.1)	
Type of TreatmentFacility	Private Tertiary Care Hospital	32 (38.1)	20,000 (10,000–50,000)	242.8 (121.42–607.1)	0.309
	Government-funded Tertiary Care Hospital	45 (53.6)	30,000 (10,000–55,000)	364.27 (121.42–667.82)	
	Private Hospitals	7 (8.3)	45,000 (45,000–120,000)	546.4 (546.4–1457)	
Experiencing Catastrophic Health Expenditure	Yes	25 (33.3)	15,000 (10,000–30,000)	182.13 (121.42–364.2)	** < 0.001**
	No	50 (66.7)	50,000 (23,750–70,000)	607.1 (288.38–849.9)	
Socioeconomic Class ^11^	Upper	1 (1.2)	1000	12.14	0.367
	Upper middle	9 (10.7)	10,000 (5000–40,000)	121.4 (60.7–485.6)	
	Lower middle	29 (34.5)	40,000 (20,000–50,000)	485.6 (242.8–607.11)	
	Upper lower	41 (48.8)	25,000 (11,000–65,000)	303.5 (133.5–789.24)	
	Lower	4 (4.8)	17,500 (6250–51,250)	212.4 (75.8–622.2)	
Health Insurance status	Insured	9 (9.7)	5000 (3000–7100)	60.71 (36.4–86.21)	** < 0.001**
	Uninsured	84 (90.3)	27,500 (10,000–50,000)	333.9 (121.4–607.1)	

*July 5 2023 exchange rate—82.3 INR₹/1 USD$

Significant values are in [bold].

**Table 4:** Financial sources met for the treatment of T1DM adolescents.

Categories	Frequency (%)	USD $	Average OOP Health Expenses /month (INR)
Own income and use saving	40 (35.3)	46.6	3849
Borrowing money (with Interest)	58 (51.3)	100.0	8264
Selling of assets	5 (4.4)	62.1	5133
Contributions or assistance from friends and relatives	10 (8.8)	62.9	5200

*July 6 2023 exchange rate—82.5 INR₹/1 USD$.

The largest proportion of participants (46%) were in the early adolescent age group (10–13 years). Fifty-nine participants were male, and 54 participants were female. The education levelof the head of the family varied from illiterate (16%)to completed high school (24%). The majority of participants were in the lower middle class (31.85%) or upper lower class (52.21%). A significant proportion of participants (88.4%) did not have health insurance. This could indicate a burden in the case of medical emergencies. The majority of adolescents (79%) were diagnosed at 1–4 years of age, 86% had controlled glycemic levels, and 48%were attending primary school.

Table 2 shows that 47.79% of the participants resided in rural areas and 52.21% resided in urban areas, and the median costs of outpatient care wereUS$36.4(IQR = US$4.8–75.9) and US$36.4(IQR = US$24.3–42.5) in rural and urban areas, respectively. Rural participants had to travel to tertiary care hospitals or clinics for T1DM treatment because the services werenot available in subcentersor primary health centers. Approximately 51.32%, 41.59%, and 7.07% of the participantsused private tertiary care hospitals, government-funded tertiary care hospitals, and private clinics, respectively, and there was a statistically significant (p = 0.004) difference in the treatment cost amongthe facilities. Of the total participants, 32.2% experienced catastrophic health expenditures. SEC wascategorized into different classes, and there wasa difference in the median cost of expenses between the lower SEC and other SECs. Only 11.59% of the participants had health insurance, while 88.4% did not. The median cost for insured participants wasUS$30.39(IQR = US$5.47–60.7), and that for uninsured participants wasUS$36.4(IQR = US$24.3–60.7). Apvalue of 0.008 was considered to indicatea statistically significant difference in the median cost between insured and uninsured participants.

Table 3 shows that among the 113 participants, 84 were hospitalized in the past 12 months, and the median costs of inpatient care wereUS$303.5(IQR = US$1214.2–607.1) and US$485.69(IQR = US$242.8–607.1) in rural and urban areas, respectively. The median costs of treatment during hospitalization wereUS$242.8 (IQR = US$121.4–607.1), US$364.2(IQR = US$121.4–667.8), and US$546.4(IQR = US$546.4–1457) in private tertiary care hospitals, government-funded tertiary care hospitals and private hospitals, respectively. Of the total participants, 33.3% experienced CHE, and there was a statistically significant difference in the median cost of expenses between the two groups, with a p value of 0.001. There wasa difference in the median cost of expenses between some socioeconomic classes. Only 11.59% of the participants had health insurance, while 88.4% did not. The median cost for insured participants wasUS$60.7(IQR = US$36.4–86.2), and that for uninsured participants wasUS$333.9(IQR = US$121.4–607.1). Apvalue of 0.001 was considered to indicatea statistically significant difference in the median cost between insured and uninsured participants.

More than50% of the financial sources for treatment were met by borrowing money with interest (58 patients, 51.3%), followed by individualincome (40 patients, 35.3%), contributions from friends and relatives (10 patients, 8.8%), and selling of assets (5 patients;4.4%).

There was a positive correlation (rvalue of 0.979) between the cost of treatment and the DSQoL score, and this correlation was statistically significant, with a *p* value < 0.001. Thehigher the DSQoL score was, the worse the quality of life and the worse the well-being of adolescents with T1DM.

The association between the DSQoL score and inpatient care cost was analyzed using a scatter plot (Fig. 1), in which the cost of treatment is shown on the X-axis and the DSQoL score is shown on the Y-axis. The relationship between the two variables wasdeterminedby alinear regression equation (Y = 0.0027x + 38.234), with a fit line R^2^of0.56 indicating a moderate fit. The coefficient x (0.002), representing the slope,indicatedthat forevery one-unit increase in the cost(x), the DSQoL (y) score increased by 0.002 units. A constant score of 38.23 representedan intercept indicating that at a cost(x) of zero, the DSQoL(y) score wasexpected to be 38.23.

The above interpretation can also be described as follows:

A scatter plot (Fig. 1) shows the relationship between the treatment cost (X-axis) and the DSQoL score (Y-axis). The linear regression equation wasY = 0.0027x + 38.234, with an R2of 0.56, suggesting a moderate fit. Eachone-unit increase in costincreased the DSQoL score by 0.002 units, starting at 38.23 points when the cost waszero.

## Response from respondents and their parents: Cost as a barrier to treatment

Overall,three themes were identified:the first theme wasthe impact of financial costs on family coping. The second themewasthe impact of financial costs on seeking care. The thirdtheme wasthe emotional burden of financial costs.

**Theme 1:** Patients 4, 7, 9, 10, and 21describedhow their regular monthly expenses were disrupted because of treatment and associated issues. Thisaffected not only the patientsbut also their families. An increase in expenses ledto challenges in meeting other financial obligations, causing strain and stress within the family.

**P 4** – *“Yes, so we went to a government hospital; we still had to pay for insulin, and weeven needed to pay for the food and travel costs.Also, the food or diet for the child wasnot available, sowe considered suicide with the child, but we have anotherfemale childand we need to survive”.*

**P 7** – *“Yes, my mother left, myfather is addicted to alcohol, and they have three children. I am the youngest, and my grandmother takes care of us; she also has to go for a coolie; then, only I will be able to get insulin.”*

**P 9—** *“Yes, I am working because of her and her treatment; my husband alone cannot bear all the expenses”.*

**P 10** – *“I am a coolie, I borrow money from finances. I want my daughter to be healthy, so we go to government hospitals, but they don’t have facilities and refer us to private hospitals.We have lost our assets, not thinking of –assets—let my life also go; I want my daughter to be healthy, which is not possible”.*

**P21**- “*In government hospitals, insulin and pens are given free of cost, even if my child is admitted.Only a few medicines are not available in the hospital and they ask us to bring few tests from outside.Even for reports, we go to a private lab, which is when my daughter was diagnosed for the first time. I had to sell my auto rickshaw for only 1.5 lakhs which were of 2 lakhs, and I had to pay 40,000 rupees for labsalone”.*

**Theme 2:** Patients 5, 42, and 57statedthat they were forced to make treatment decisions based on economic considerations. This indicatedthat treatment expenses hinderedpatients’abilitiesto seek necessary treatment, leading to compromised health outcomes.

**P5Mother**- *“Yes, the midnight sugar level was high; we have faced many problems taking my child to the hospital, without money for travel. We requested that we pay an automobile driver later; God helped us on that day and the driver did not take any money. Theydropped us atthe hospital, and my child survived because of the staff nurses working that day”.*

**P42 Mother- “** *I am handicapped, my husband left me 8 years ago, and my 10-year-old son is suffering from diabetes. I stay with my brother’s family, and last month my son’ssugar level shotup suddenly despite me giving him insulin.Hewas not able to get up from his bed, so I took him to hospital. I had to beg for money from my brother to pay for treatment, as I get a handicap pension of 600 rupees per month”.*

**P57 Father-*“****My daughter is 15 years old, and I want her to be healthy.We have compromised with everything madam: we don’t buy new clothes, nor do we go to hotels, we don’t buy fruits or dried fruits because I have to save money for my daughter's treatment.She is like a bubble on water, I can’t say what may happen at anytime.My daughter resembles my mother, she is so beautiful but I don’t know why God gave her this disease. I will pray forany miracle to happen and my daughter should be free from the disease*”.

**Theme 3:** Patients 6, 14, and 18 expressed emotional impact of treatment expenses. Many participants expressed feelings of suicidal thoughts, frustration, and hopelessness, mainly because of limited access to T1DM-related health services, necessary supplies, and medication, which may significantly impact QOL.

**P 6 Mother** *- “Yes, Vedavathi and Varshini (names changed) are twins and both have diabetes, so we need to at least give them insulin ……we are unable to give it sometimes, when their sugar levels suddenlyshotup.We may miss meals, not their insulin. Can we obtain free insulin somewhere?”.*

## P 14–

*“Yes, so we went to a government hospital; we still had to pay for insulin, and weeven needed to pay for the food and travel costs.Also, the food or diet for the child wasnot available, so we considered suicide with the child, but we have anotherfemale childand we need to survive”.*

**P18 –** ***Father*** *- “I earn around ten to eleven thousand rupees per month* ***.*** *If I calculate our expenses for my daughter, the cost isabout 8 rupees for insulin injection, 550 rupees for strips to check her sugar levels, whichwe need to check daily before giving her insulin, 400 rupees for insulin vials, and we need 4 per month.Once we visit the hospital for auto charges and bus fare it isapproximately 400rupees, a lab test costsapproximately 900–1000 rupees, a doctor’s consultation costs 400 rupees). It will be approximately 3500–4000 rupees; last time she was admitted because of increased sugar levels, we had to stay for 10 days in the hospital and had to pay approximately 90,000rupees. Sometimes I pray to God; poor people should not get any disease and experiencehell on earth.”*

## Discussion

T1DM is a chronic illnessthat requires regular medication in the form of insulin. Managing adolescents with T1DM is challenging for families both socially and economically and affects quality of life. This study investigated the cost of illness and its impact on quality of life.

The average median health expenses were 3000(400–6250) and 3000(2000–3500) rupees per month for the rural and urban participants, respectively, in the present study. There is no accessibility of insulin in subcentersor primary health care centers. Patients hadto travel to public or private tertiary care hospitals or receive treatment in private clinics, which increasedtheir out-of-pocket expenses (OOPEs), especially for the rural population. Some NGOs help these patients at tertiary care hospitals by providing free insulin. In one of the studies performed by the GOI, the monthly expenditures for diabetes treatment in 2017 were 2,893 and 4,162 rupees, respectively, for rural and urban participants (T1DM and T2DM)in 25 states and union territories^16^. Ina study conducted in India, the median average direct and indirect costs of diabetescare were 25,391 and 4970 rupees annually, respectively^17^. According to a National Health Accounts (2019) report^18^**, **OOPEsdecreased by 16%, from 64.2% to 48.2%, following the launch of the Ayushman Bharath Scheme and several such actions. Even per capita, OOPEsalso decreased by 8% from 2,366 rupees in 2013–14 to 2155 rupees in 2018–19. There was a slight difference betweenthe expenses in our study (3000 rupees) and National Health estimates (2155 rupees), as their data collection was in the year 2018, and our data collection occurred in2023^18^.Ayushman Bharath mainly focused on secondary and tertiary care.The importance of primary health care services through HWCs was also recognized, but T1DM-specific management and services are not accessible forpatients, especially the rural population^19^. OOPEsare greater in rural areas than in urban areas. Some NGOs help T1DM patients who belong to BPL families by providing free life-saving medicine and insulin, glucometers, strips, and pens,which is a kind of public‒private partnership. Examples include theEDRT of Novo Nordisk^20^ and theNityaasha Foundation^21^.The tertiary care hospitals (public and private) where we collected data collaborate with these NGOs, butsome tests are not conducted free of cost, some drugs are not available in government-funded hospitals, anddoctors prescribesome medications from outside, which leads to OOPEs.

The proportion of patients with catastrophic health expenditure (CHE) was 32.7% in our study. Similar findings were observed inthe National Health Services Survey (NHSS) conducted in 2013, 2018 to determine the incidence of CHE among poorer households, elderly persons, and individuals who visit private health clinics for treatment^22^. Similarly, in 2013 and 2018, a study in China using the National Health Services Survey (NHSS) revealed that the incidence of CHE in urban areas was 27.50%(2013) and 34.52%(2018) and that in rural areas was 21.72%(2013) and 35.89%(2018)^23^.

In the present study, only approximately 12% of the households had health insurance, there was a statistically significant difference in the average median cost amongsocioeconomic classes.A study showed that households with a low socioeconomic status had a greater proportion of OOPEsand that insurance reduces the risk of catastrophic health expenditures^23^.

**In the present study, **more than 50% of the financial sources for treatment were met by borrowing money with interest (58 patients, 51.3%), followed by individualincome (40patients, 35.3%), contributions from friends and relatives (10patients, 8.8%), selling of assets (5patients, 4.4%). A study conducted in India among rural and urban populations showed that 58% of the patients borrowed money to pay for treatment, 42% of hospitalized patients received money from friends and relatives. Approximately 60% of rural households, 40% of urban households sold or relied on assets and borrowed money for treatment when patients were hospitalized^14^. In systematic review studies,the results showed high OOPEs, CHEs, and impoverishment among noncommunicable disease patients, selling assets and borrowing money were common coping strategies adopted^24^.

There wasa positive relationship between the cost of illness and diabetes-specific quality of life; the higher the cost was, the worse the quality of life among the type 1 diabetic adolescents in our study. A study conducted in tertiary care hospitals with 59 type 1 diabetic patients showed that treatment expenses and glycemic control were negatively correlated^8^. There was poor quality of life and an increased burden on individuals with psychosocial illness who spent less on diabetestreatment^8^. The costof treatment and glycemic control wererelated, but the cost andQOL among adolescents with T1DMdepended not only on the cost of treatment but also on education level, income, accessibility to health care services, clean and safe housing, and diet^25^. In a study conducted among cancer patients, it was observed that there was a correlation between the cost of chemotherapy and QOL;the cost increased with each round of chemotherapy, the QOL increased with each chemotherapy cycle^26^.

## Conclusion

Cost acts as a barrier to treatment, and disease treatment has become a burden for patients and their parents. Significantly lower health expenses were observed among participants who had health insurance. Hence, awareness about government health insurance schemes should be increased. Health services related to T1DM are not accessible in rural areas;patientsmust travel to cities or tertiary care hospitals, leading to OOPEsand catastrophic health expenses. The health expenses of the patients in our study weregreater than their monthly incomes, which wasmore evident among families with low socioeconomicstatus. T1DM health services should be made available in subcenters and primary health care facilities to reduce travel expenses for individuals and their families, especially for the rural population. The Diabetes-specific Quality of Life (DSQoL) Questionnaire has not been validated for the Indian population.

### Supplementary Information

Supplementary Information 1.

Supplementary Information 2.

Supplementary Information 3.

## Data Availability

All data generated or analyzed during this study are included in this published article [as supplementary information files].
