# Supplementary material for: Relationship between the cost of illness and quality of life among adolescents with type 1 diabetes—a mixed method study
Source: Sci Rep. 2024 Jun 11;14:13403. doi: 10.1038/s41598-024-63536-4 (PMC11167017; doi:10.1038/s41598-024-63536-4)
Supplement: Supplementary file 1 — Supplementary Information 1. [file 41598_2024_63536_MOESM1_ESM.docx]

Annexure 1:

Scoring of DSQoL:

Higher percentages indicates= worse diabetic quality of life, Worst wellbeing

| **Subscales** | **DSQoL Scale Question #** | **Subscale Score Range** |
| --- | --- | --- |
| **Social aspects** | (SA) 4, 6, 9, 15, 20, 28, 31, 34, 39, 40, 44, 47, 54, 57, 64 | SA total/75*100 |
| **Dietary restrictions** | (DR) 2, 23, 24, 29, 37, 42, 50, 60, 40 | DR total/40*100 |
| **Fear of hypoglycemia** | (FH)10, 22, 27, 36, 41, 45, 46, 48, 52, 58 | FH total/50*100 |
| **Physical complaints** | (PC) 3, 11, 16, 19, 21, 25, 30, 32, 35, 51, 61 | PC total/55*100 |
| **Anxiety about future** | (AF) 5, 7, 8, 14, 18, 26, 38, 43, 49, 59, 62 | AF total/55*100 |
| **Daily hassles** | (DH) 1, 12, 13, 17, 33, 53, 55, 56, 63 | DH total/45*100 |
| **Total score** | All questions 1 - 64 | Grand total/320*100 |
